# Emerging roles of ferroptosis in pulmonary fibrosis: current perspectives, opportunities and challenges

**DOI:** 10.1038/s41420-024-02078-0

**Published:** 2024-06-24

**Authors:** Yixiang Hu, Ying Huang, Lijuan Zong, Jiaxin Lin, Xiang Liu, Shipeng Ning

**Affiliations:** 1 Department of Clinical Pharmacy, The Affiliated Xiangtan Center Hospital of Hunan University, Xiangtan, 411100 China; 2 Zhongshan Hospital of Traditional Chinese Medicine Afflilated to Guangzhou University of Chinese Medicine, Zhongshan, 528400 China; 3 Department of Rehabilitation Medicine, Zhongda Hospital of Southeast University, Nanjing, 210096 China; 4 Department of Breast Surgery, The Second Affiliated Hospital of Guangxi Medical University, Nanning, 530000 China

**Keywords:** Cell death, Respiratory tract diseases, Stress signalling

## Abstract

Pulmonary fibrosis (PF) is a chronic interstitial lung disorder characterized by abnormal myofibroblast activation, accumulation of extracellular matrix (ECM), and thickening of fibrotic alveolar walls, resulting in deteriorated lung function. PF is initiated by dysregulated wound healing processes triggered by factors such as excessive inflammation, oxidative stress, and coronavirus disease (COVID-19). Despite advancements in understanding the disease’s pathogenesis, effective preventive and therapeutic interventions are currently lacking. Ferroptosis, an iron-dependent regulated cell death (RCD) mechanism involving lipid peroxidation and glutathione (GSH) depletion, exhibits unique features distinct from other RCD forms (e.g., apoptosis, necrosis, and pyroptosis). Imbalance between reactive oxygen species (ROS) production and detoxification leads to ferroptosis, causing cellular dysfunction through lipid peroxidation, protein modifications, and DNA damage. Emerging evidence points to the crucial role of ferroptosis in PF progression, driving macrophage polarization, fibroblast proliferation, and ECM deposition, ultimately contributing to alveolar cell death and lung tissue scarring. This review provides a comprehensive overview of the latest findings on the involvement and signaling mechanisms of ferroptosis in PF pathogenesis, emphasizing potential novel anti-fibrotic therapeutic approaches targeting ferroptosis for PF management.

## FACTS

- Ferroptosis is a novel regulated cell death mechanism characterized by intracellular iron overload and lipid peroxidation.
- Ferroptosis plays a crucial role in the development of pulmonary fibrosis, driving macrophage polarization, fibroblast proliferation, and ECM deposition.
- Targeting ferroptosis presents new promising targets for halting the progression of pulmonary fibrosis.
- A thorough investigation of the molecular mechanisms driving ferroptosis and its regulatory factors significantly advances our knowledge of the pathogenesis of pulmonary fibrosis.

## OPEN QUESTIONS

- What are the mechanisms and significance of ferroptosis in pulmonary fibrosis?
- What are the implications of the interaction between the ferroptosis pathway and other cell death pathways for the initiation and advancement of pulmonary fibrosis?
- The potential of ferroptosis-related molecules as biomarkers for the early detection, diagnosis, prognosis, and treatment of pulmonary fibrosis warrants exploration.
- In clinical treatment, how to effectively combine ferroptosis inhibitors with anti-fibrotic drugs to achieve optimal therapeutic outcomes?

## Introduction

Pulmonary fibrosis (PF) is a chronic, progressive, and fatal interstitial lung disease (ILD) associated with loss of alveolar gas exchange function and excessive deposition of extracellular matrix (ECM) [1], leading to irreversible progressive lung scar formation. However, the etiology of PF remains elusive in the majority of patients and is classified as idiopathic pulmonary fibrosis (IPF), characterized by progressive exacerbation of dyspnea, persistent dry cough, restrictive ventilation dysfunction, and ultimately culminating in respiratory failure-induced mortality [2, 3]. The incidence of IPF ranges from 0.9 to 9.3 cases/100,000 per year in Europe and North America, and from 3.5 to 13.0 cases/100,000 per year in Asia and South America [4]. Of note, the COVID-19 pandemic and subsequent global transmission have resulted in a cumulative total of over 400 million confirmed cases, with PF emerging as one of the major long-term complications [5–7]. Additionally, PF is more prevalent among the elderly population, typically presenting between the ages of 50–70 years with a higher prevalence in men than women [4]. The median survival in PF is limited to 3–5 years, with increasing morbidity and mortality rates observed annually [8]. In 2014, the FDA granted approval for two drugs, pirfenidone and nidazanib, as therapeutic options for PF treatment [9]. However, these therapies primarily aim to decelerate disease progression without effectively reversing fibrosis or significantly improving overall survival, while potentially inducing adverse effects such as gastric and intestinal bleeding, along with severe diarrhea [10]. Apart from lung transplantation, no current interventions exist that can effectively alter the natural course of PF [11]. Consequently, the active pursuit of anti-fibrotic therapeutic agents holds significant importance.

Repeated injury to alveolar epithelial cells (AECs) serves as a pivotal instigator in the initiation of fibrosis [12]. Persistent cellular damage provokes an inflammatory response that recruits innate immune cells (particularly macrophages) to the site of injury and elicits the release of pro-fibrotic factors, such as transforming growth factor β (TGF-β) and α-smooth muscle actin (α-SMA) [13]. These pathogenic events stimulate fibroblasts to proliferate and transform into myofibroblasts. In fact, myofibroblasts play a crucial role in fibrosis by overproducing ECM components, such as laminin (LN), matrix metalloproteinases (MMPs), and type I, III, and IV collagen proteins, thereby contributing to the thickening and stiffening of pulmonary tissue [14, 15]. Emerging evidence suggests that imbalanced oxidative/antioxidant-induced ferroptosis in the lung plays a crucial role in the progression of PF [16]. Inhibiting lipid peroxidation by applying ferroptosis inhibitors has proven effective in halting fibrogenesis.

Ferroptosis is a newly discovered iron-dependent regulated cell death (RCD) mode that was officially named by Dixon and colleagues in 2012 [17]. This novel RCD mechanism does not exhibit typical apoptotic characteristics, such as nuclear fragmentation and cysteine asparaginase activation [18]. The hallmarks of ferroptosis include lipid peroxide accumulation, iron overload, and excessive reactive oxygen species (ROS) production [19]. Iron overload-induced ferroptosis has been increasingly recognized as a critical contributor to the pathogenesis of multiple fibrotic diseases, including hepatic fibrosis [20], renal fibrosis [21], radiation-induced intestinal fibrosis [22], myocardial fibrosis [23], and PF [24]. During the progression of PF, various induction factors, such as cigarette smoke [25], bleomycin (BLM) [24], paraquat (PQ) [26], silicosis [27], and PM2.5 [28], have been shown to trigger the initiation of ferroptosis, leading to fibroblast-to-myofibroblast differentiation and pro-fibrotic factors release.

The aim of this review was to investigate the roles of lipid peroxidation and ferroptosis in the pathogenesis of PF. Additionally, we discuss the therapeutic potential of targeting ferroptosis for PF treatment and propose several potential predictive indicators and treatment strategies.

## Molecular insights into the pathological process of PF

**Fig. 1:**
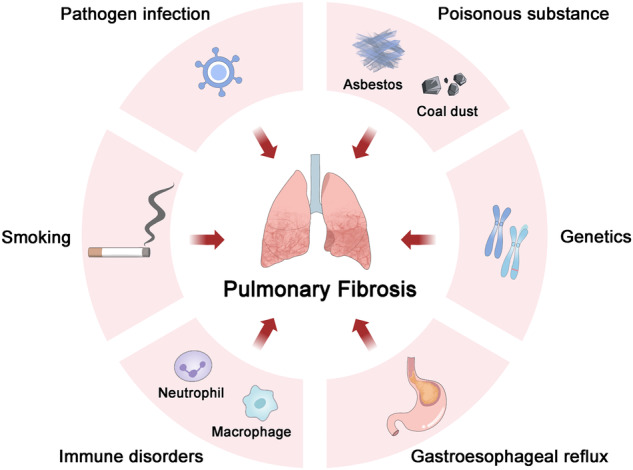
Risk factors of PF. Numerous risk factors have been implicated in the development of PF, including exposure to tobacco smoke, gastroesophageal reflux, viral and bacterial infections (such as COVID-19), toxic substances (such as asbestos, silicon dioxide, PM2.5, and PQ), genetic variations, and immune disorders.

Lungs serve as vital organs for the gaseous exchange in mammals and contain the most extensive epithelial surface in direct contact with the external environment. However, ambient air harbors a multitude of particles encompassing pollutants, microorganisms, and oxidants that have the potential to damage the delicate structure of the alveolar epithelium [5, 29]. The persistent inflammatory response in the lungs is recognized as a precursor to fibrosis. Numerous factors contribute to PF, including exposure to tobacco smoke, gastroesophageal reflux, viral and bacterial infections, silica dust inhalation, genetic variation, and immune disorders (Fig. 1). If risk factors such as silica and asbestos cannot be eliminated promptly, acute inflammation may progress into chronic inflammation, resulting in aberrant wound healing responses and fibrosis [30]. Although therapeutic strategies targeting the immune-inflammatory response (e.g., corticosteroids and immunosuppressive drugs) are effective in non-PF interstitial lung disease, they have no discernible impact on the fibrotic process in PF [31]. Therefore, in order to impede the progression of fibrosis, it is necessary to explore other novel therapeutic strategies.

**Fig. 2:**
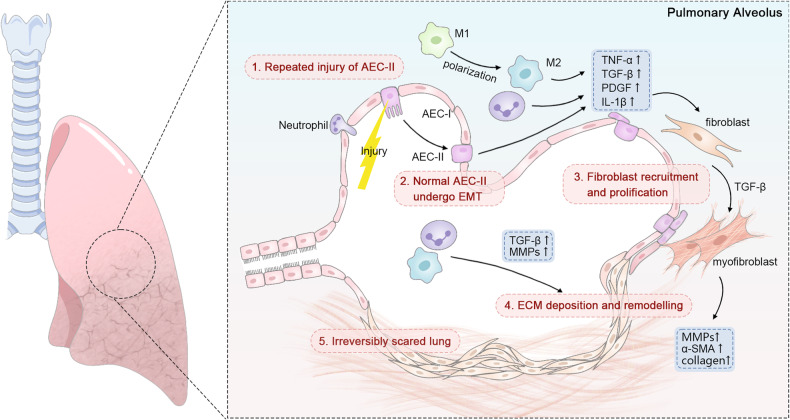
Molecular insights into PF pathophysiology. Repeated injury to AECs leads to chronic inflammation, which is considered to be the initiating event of PF, followed by ACE-II EMT, neutrophil infiltration, and macrophage polarization. A multitude of pro-fibrotic mediators (such as TGF-β, IL-1β, TNF-α, and PDGF) are then released, leading to fibroblasts proliferation and the EMT of AECs. These molecular events progressively triggering macrophage polarization, fibroblast proliferation, and myofibroblast activation. Subsequently, the over-synthesis of ECM components by myofibroblasts contributed to reduced lung compliance and ultimately irreversible PF.

In patients with PF, the progression of fibrosis is believed to be influenced by three primary factors: excessive epithelial damage caused by inhaled pollutants, senescence, and genetic susceptibility [32]. The initial event is thought to involve damage to the AECs lining the air sacs of the lungs, which can be caused by a variety of factors including viral infections, chemical agents, radiation exposure, environmental toxins or genetic predisposition [33]. The alveolar epithelium is primarily composed of type I AECs (AEC-I) and type II AECs (AEC-II). Notably, the balance of functionality between AEC-I and AEC-II is critically involved in the pathogenesis of PF. Under normal physiological conditions, AEC-II is primarily responsible for producing pulmonary surfactant to maintain the surface tension and stability of alveoli [34]. Additionally, AEC-II notably contribute to tissue repair and regeneration by exhibiting the capability to differentiate into AEC-I and promote the regeneration of damaged alveolar structures [35]. However, in response to lung injury and stress, the abnormal activation and excessive proliferation of AEC-II can lead to persistent inflammatory responses and the initiation of fibrotic pathologies [36]. This damage triggers the recruitment of fibroblasts and immune cells such as macrophages and neutrophils to injury site. Subsequently, multiple pro-fibrotic factors (e.g., chemokines, proteases, and TGF-β) are released, leading to the transition of AEC-II into a mesenchymal phenotype through the process of epithelial-mesenchymal transition (EMT), ultimately resulting in basement membrane destruction [8, 37]. Macrophages, constituting ~70% of the immune cells in the pulmonary, play a critical role in the airway remodeling process in PF [38]. The polarization of macrophages into M1 or M2 phenotypes is modulated by stimuli and signals from the inflammatory microenvironment [39]. During the pathological progression of PF, an excessive polarization of macrophages towards the M1 phenotype leads to epithelial cell demise, while uncontrolled infiltration of M2 macrophages in the lungs results in the release of a multitude of profibrotic cytokines, including IL-1β, tumor necrosis factor-alpha (TNF-α), TGF-β, platelet-derived growth factor (PDGF), and fibroblast growth factor receptor (FGFR) [40–42]. These molecular events stimulate fibroblast proliferation and differentiation into myofibroblasts, a specialized subtype capable of contraction, which play a crucial role in the wound healing process [43]. During chronic inflammation, myofibroblasts are characterized by high matrix remodeling activity and the ability to generate abundant ECM components (e.g., α-SMA, collagen, and MMPs) [44, 45], leading to the thickening and sclerosis of lung tissue, ultimately culminating in PF (Fig. 2).

## Lipid peroxidation participates in PF

Recent studies have elucidated the critical role of lipid peroxidation in the pathogenesis of PF. Excessive ROS production or depletion of antioxidant capacity leading to heightened oxidative stress has been implicated in fibrotic development across multiple organs, including the heart, liver, and lungs [46]. Of note, oxidative stress induces lipid impairment primarily affecting cell membrane lipids such as phospholipids and cholesterol, thereby significantly impacting the permeability and fluidity of the lipid bilayer [47]. This mechanism has been widely recognized as a cause of cell damage by altering the composition of cell membranes [48]. Lipid peroxidation leads to the generation of harmful byproducts, such as 4-hydroxy-2-nonenal (4-HNE) and malondialdehyde (MDA) [47, 49, 50]. Importantly, the upregulation of TGF-β expression by 4-HNE in macrophages suggests the presence of a detrimental feedback loop between lipid peroxidation and excessive TGF-β production [51].

Lipid peroxidation proceeds through three distinct phases: initiation, proliferation, and termination. During the initiation phase, lipid free radicals are formed as initiators for the peroxidation chain reaction [52, 53]. In the proliferation phase, these lipid radicals react with molecular oxygen to generate lipid peroxyl radicals. Subsequently, lipid peroxyl radicals further interact with fatty acids leading to the production of either lipid radicals or lipid hydroperoxides. Throughout this period, the presence of unstable lipid hydroperoxide (LOOH) results in the generation of various secondary products such as 4-HNE, propionaldehyde, hexenal, MDA, and acrolein [47]. Finally, during the termination phase non-radical species are formed where antioxidants play a crucial role by acting as hydrogen atom donors to effectively terminate the peroxidation chain reaction. Several pathways, including catalase and superoxide dismutase (SOD), glutathione (GSH), and glutathione peroxidase (GPX), can inhibit lipid peroxidation [54]. GPX4, a selenoprotein, directly contributes to the reduction of peroxidized phospholipids in cell membranes for maintaining redox homeostasis [55–57]. Additionally, vitamin E and vitamin C possess the capability to prevent lipid peroxidation by scavenging free radicals [58].

Imbalanced oxidant-antioxidant dynamics have been observed in the lungs of patients with PF [59]. Rahman et al. reported elevated levels of lipid peroxidation products in bronchoalveolar lavage fluid (BALF) and plasma samples obtained from individuals diagnosed with PF [60]. Furthermore, a recent study unveiled a significant upregulation of 4-HNE expression in lung fibroblasts isolated from PF patients, concomitant with a concurrent reduction in GPX4 levels and an elevation in 4-HNE expression observed in an in vivo model of bleomycin (BLM)-induced PF [59]. Notably, the administration of Trolox effectively mitigated BLM-induced lipid peroxidation and attenuated the progression of PF [59]. Konoh and colleagues collected BALF samples from 34 patients diagnosed with PF and observed a significant correlation between elevated ethane accumulation levels and a poorer prognosis [61]. On the other hand, alterations in antioxidants have also been detected in PF lungs [61]. Evidence indicates that fibroblastic lesions in PF patients exhibit a downregulation of sulfiredoxin-1 and nuclear factor erythroid 2-related factor 2 (Nrf2) [62]. Additionally, peroxiredoxin-1, an antioxidant protein that protects cells from oxidative damage induced by ROS, was also found to be reduced in BALF from PF patients [63]. As an FDA-approved anti-fibrotic medication for the treatment of IPF, pirfenidone effectively impedes the progression of BLM-induced PF by upregulating the expression levels of Nrf2, HO-1, and GPX1 [64]. Intriguingly, recent mechanistic studies have revealed that the reduction in transition metal levels may contribute to the state of oxidative stress [65, 66]. Remarkably lower concentrations of chromium, zinc, ferrous, and nickel ions were detected in BALF from IPF patients [67]. Accumulating evidence suggests that zinc acts as an antioxidant and plays a crucial role in the structural remodeling of lung tissue through its mediation of metalloproteinases [68]. Furthermore, manganese is involved in the production of specific antioxidants within pulmonary tissue, such as Mn-SOD located within mitochondria [69].

## Overview of ferroptosis and its signal transduction

RCDs are essential for numerous biological processes, such as the maintenance of normal homeostasis and elimination of detrimental stimuli [70]. These distinct RCD subroutines, such as apoptosis, autophagy, pyroptosis, cuproptosis, and ferroptosis, exhibit unique characteristics and displaying significant overlap and crosstalk [71].

**Fig. 3:**
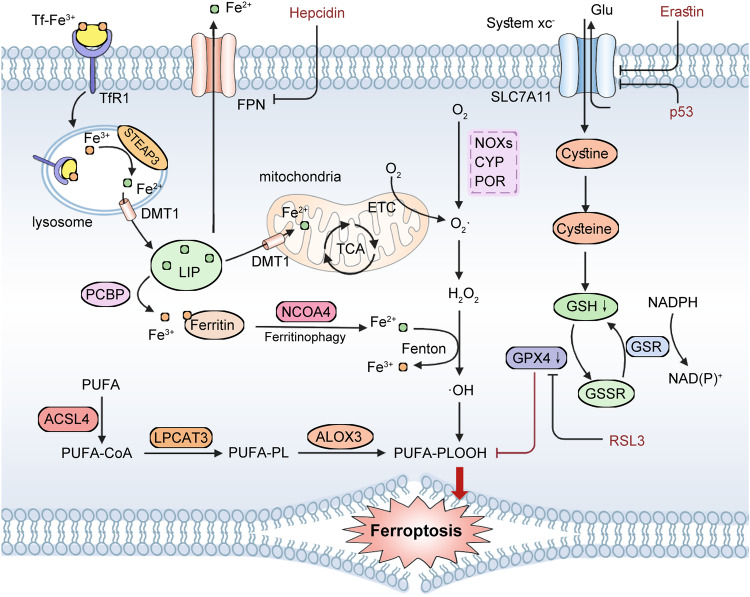
Molecular mechanisms of ferroptosis. TfR1 mediates the endocytosis of Tf-Fe^3+^ into lysosomes for iron uptake. Subsequently, Fe^3+^ is converted to Fe^2+^ by STEAP3 and transported into the labile iron pool via DMT1. Additionally, Fe^2+^ can be sequestered by ferritin after being converted to Fe^3+^ through PCBP-mediated processes. In cases of intracellular iron deficiency, NOCA4-mediated ferritinophagy restores the levels of available iron ions. However, when free ferritin ions enter mitochondria via DMT1, they induce oxidative stress. The Fenton reaction, facilitated by iron, generates substantial amounts of reactive oxygen species (ROS), leading to lipid peroxidation primarily targeting polyunsaturated fatty acids (PUFAs). PUFA peroxidation necessitates the involvement of ACSL4 and LPCAT3 enzymes and ultimately triggers ferroptosis. Cyst(e)ine/GSH/GPX4 axis regulates ferroptosis by mitigating the detrimental effects of lipid peroxidation through its reduction back to lipids. Impaired GPX4 function or inhibition of system xc- activity along with depleted GSH levels result in lipid peroxide accumulation and subsequent ferroptotic cell death.

Ferroptosis is a recently discovered form of RCD that arises from iron-dependent lipid peroxidation and excessive ROS production (Fig. 3). Its distinct morphological, biochemical, immunological, and genetic characteristics set it apart from other RCD mechanisms [72]. The cellular morphological features of ferroptosis primarily include mitochondrial cristae shrinkage, increased density of the mitochondrial bilayer membrane, a normal-sized nucleus without pyknosis, and loss of cell membrane integrity leading to lysis [73]. Mechanistically, the induction of ferroptosis is intricately associated with an imbalance between the oxidative and antioxidant systems within the organism. It should be emphasized that not all ROS equally contribute to the occurrence of ferroptosis, despite its initiation being attributed to oxidative damage [53]. Notably, iron-dependent ROS generation emerges as the primary catalyst for lipid peroxidation-induced ferroptosis, implying that distinct molecular mechanisms are indispensable for its initiation and execution.

Lipids containing diallyl carbon and polyunsaturated fatty acids (PUFAs) are highly susceptible to lipid peroxidation [74]. By employing whole-genome haploid screening and CRISPR-Cas9 technology, two pivotal drivers of ferroptosis were identified: Acyl-CoA synthetase long-chain family member 4 (ACSL4) and lysophosphatidylcholine acyltransferase 3 (LPCAT3), both of which function as membrane remodeling enzymes [75–77]. ACSL4 catalyzes the addition of CoA to polyunsaturated fatty acids (PUFAs). In conjunction with LPCAT3, it synthesizes phospholipids containing polyunsaturated fatty acids (PUFA–PLs), which are subsequently incorporated into the cell membrane and trigger ferroptosis [78–80]. Importantly, the accumulation of oxidized PUFAs at the cell membrane is essential for promoting ferroptosis, resembling a lethal buildup of lipid peroxides [81]. Lipid peroxides are primarily generated intracellularly through two processes: enzyme-catalyzed lipid peroxidation and the Fenton reaction induced by free iron ions. Enzymes involved in enzyme-catalyzed lipid peroxidation include the lipoxygenase LOX family, specifically Arachidonic acid 15-lipoxygenase (ALOX15), NADPH-cytochrome P450 reductase (POR), and NADH-cytochrome b5 reductase (CYB5R1) [82]. However, the depletion of ALOX15 does not rescue the ferroptosis induced by GPX4 loss [83]. Iron acts as an electron carrier in Fenton reactions, functioning as a redox catalyst and generating ROS [84, 85]. Perturbation of iron homeostasis leads to elevated levels of intracellular free ferrous iron, which reacts with peroxides to produce ferric ions and peroxyl radicals, consequently resulting in a substantial increase in ROS production [86]. Glutathione plays a crucial role as an indispensable antioxidant and scavenger of free radicals within cells, predominantly existing in its reduced form as GSH and oxidized glutathione disulfide (GSSG) [87]. Cystine serves as the primary source for GSH synthesis, obtained from the extracellular environment through the cystine-glutamate antiporter solute carrier family 7 member 11 (SLC7A11), also known as system xc^-^ [88]. Inhibition of system xc^-^ impairs cystine uptake, resulting in diminished GSH levels that subsequently reduce the activity of membrane lipid repair enzyme GPX4 and compromise cellular antioxidant capacity [78].

## Iron Homeostasis in The Lungs

**Fig. 4:**
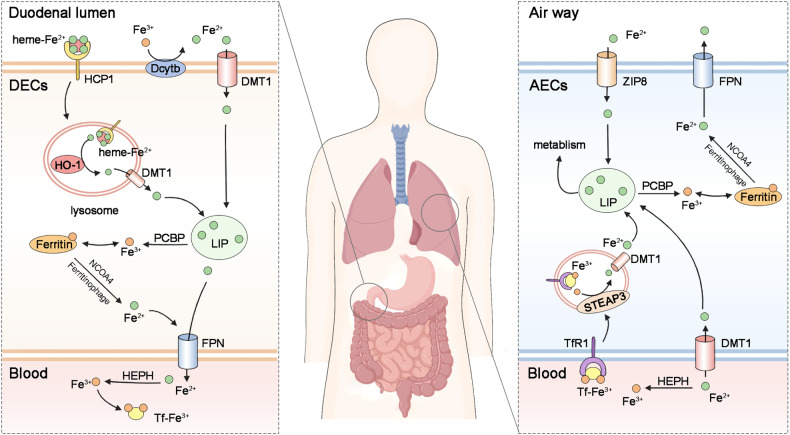
Iron homeostasis in the lungs. The iron present in foods primarily exists as heme-Fe^2+^ and nonheme iron (Fe^3+^). Fe^3+^ is reduced by Dcytb in the brush-border membrane and subsequently transported into enterocytes via DMT1. Heme-Fe^2+^ is absorbed and degraded within enterocytes by HO-1. Once exported through FPN, Fe^2+^ undergoes rapid conversion to Fe^3+^ by HEPH and binds to transferrin for circulation. The majority of Fe^3+^ is bound to transferrin, which is taken up by TfR1 at the surface of AECs, followed by reduction of Fe^3+^ to Fe^2+^ by STEAP3 and export into the labile iron pool (LIP) in the cytosol via DMTI. ZIP8 predominantly localizes at the apical surface of AECs, facilitating transport of non-transferrin-bound iron from the airways into AECs. Ferritin serves as a large iron storage site regulated by PCBP. Under conditions of iron deficiency, NCOA4 mediates ferritinophagy to increase intracellular iron levels.

Iron metabolism plays a crucial role in the pathogenesis of lung diseases. Excessive intracellular iron, particularly Fe^2+^, triggers lipid peroxidation via the Fenton reaction, resulting in ferroptosis and concomitant generation of a substantial amount of ROS [89]. In recent years, numerous studies have elucidated the involvement of ferroptosis in fibrosis across various organs, and administration of ferroptosis inhibitors has demonstrated certain protective effects [20, 21, 90]. In this chapter, we focus on delineating the roles of iron metabolism in PF (Fig. 4).

Iron is an indispensable trace element in the human body, serving multiple functions including oxygen transportation, cellular respiration, and DNA synthesis [91–94]. Under physiological conditions, the body exerts stringent control over iron uptake to prevent excessive accumulation [95]. The dietary iron absorbed through the duodenum is transported into intestinal cells, where it is exported via ferroportin (FPN) and subsequently binds to transferrin [96]. As a carrier of iron, transferrin is captured by intracellular transferrin receptor 1 (TFR1) through endocytosis. Subsequently, iron is released from transferrin and undergoes reduction to its divalent form by lysosomal reductases before being transported into the cytosol via divalent metal transporter 1 (DMT1) and transient receptor potential mucolipin ½ (TRPML1/2), ultimately entering the cellular labile iron pool (LIP) [97]. Within the LIP, iron binds to ferritin for storage until it is required for other biological activities. Nuclear receptor coactivator 4 (NCOA4) is a crucial molecule for maintaining intracellular and systemic iron homeostasis by specifically recognizing and facilitating the autophagic degradation of ferritin, leading to the release of ferric ions into the unstable iron pool. This process is commonly referred to as ferritin autophagy [98]. FPN transport functions as the exclusive mechanism for extracellular iron export. Systemic regulation of iron metabolism primarily occurs in the liver, where hepcidin binds to FPN, leading to its degradation and inhibition of iron export, thereby contributing to intracellular iron accumulation [99]. During hypoxia or instances of inadequate dietary intake, a decrease in hepcidin levels results in elevated circulating levels of bioavailable iron [100].

Due to prolonged exposure to air rich in particulate matter, infectious pathogens, and oxides, the iron homeostasis in the lungs exhibits unique characteristics (Fig. 4). Indeed, the concentration of ferritin in the pulmonary tissue surpasses that observed in other organs, indicating a direct interplay between lung tissue and exogenous iron from the environment [101]. Noteworthy, iron is primarily transported to the lungs from other organs via FPN, while a small fraction exists in a free form in the plasma. Following exposure to cigarette smoke, there was an observed increase in both iron and ferritin concentrations within rat lungs [102]. A recent study revealed that AECs and alveolar macrophages primarily internalize transferrin-bound iron through TFR1-mediated endocytic pathways, while also partially utilizing the DMT1 transport protein for iron uptake [103]. Zhang et al. reported that Zinc transporter protein ZIP8 (also known as SLC39A8), which exhibits its highest expression levels in the lung [104], is predominantly localized at the apical surface of AECs and facilitates transportation of non-transferrin-bound iron from airways into AECs [105]. Another study revealed that FPN is predominantly expressed at the apical surface of human lung airway epithelial cells and appears to play an essential role in iron detoxification [106]. Although hepcidin primarily originates from hepatic sources, an in vitro study revealed that interferon-γ (IFN-γ) regulates hepcidin expression in AECs without significantly impacting iron transport in these cells or alveolar macrophages [107]. Notably, intracellular labile iron (Fe^2+^) within lung tissue can generate numerous ROS via the Fenton reaction, thereby supplying ample raw materials for lipid peroxidation and ferroptosis, consequently triggering the development of PF [108]. Collectively, iron homeostasis is essential for maintaining the physiological function of the lungs. It might be a promising strategy to assess the risk and severity of PF by detecting iron homeostasis.

## Ferroptosis Participates In The Pathogenesis Process of PF

With an in-depth understanding of the mechanisms of ferroptosis, a growing body of research has revealed that ferroptosis plays a critical role in the pathological process of PF [24, 109]. Current evidence demonstrated that elevated levels of iron and iron-related proteins in fibrotic lung tissues, suggesting a disturbance in iron homeostasis [24]. Increased levels of ROS and LIP not only occur in fibrotic tissues but also act as mediators in regulating the onset of fibrosis [19]. Furthermore, the reduction of exogenously supplied ferritin during EMT inhibits the development of fibrosis. The decreased expression or activity of GPX4 has been identified in fibrotic lung tissue [59]. Erastin, a ferroptosis inducer, promotes TGF-β1-triggered fibroblast-to-myofibroblast differentiation in PF models in vitro by increasing lipid peroxidation and suppressing GPX4 expression. Zhuo et al. reported that the administration of bleomycin and LPS induces ferroptosis in lung epithelial cells, contributing to the progression of PF [24]. Stimulation with TGF-β upregulated the expression of transferrin receptor protein 1 (TFRC) in both human lung fibroblast cell lines and primary lung fibroblasts of mice [24]. This led to elevated levels of intracellular Fe^2+^, consequently facilitating the fibroblast-to-myofibroblast transition during the later stages of fibrosis. Interestingly, TFRC knockout mice exhibited decreased symptoms of PF following induction with bleomycin [24]. Another study showed that GPX4 and FSP1 (ferroptosis suppressor protein 1) collaborate to regulate ferroptosis in AEC-II cells in PF. The methylation regulator UHRF1, which is upregulated in mouse models of PF, promotes the development of PF by epigenetically repressing the GPX4 and FSP1 genes [110].

Several molecular mechanisms are involved in the association between ferroptosis and PF. The TGF-β/Smad signaling pathway is a canonical pathway that regulates the progression of PF. Evidence indicates that using recombinant adeno-associated virus AAV9 in combination with a TGF-β/Smad inhibitor can effectively mitigate silicosis-induced PF through ferroptosis inhibition [111]. Recently, it has been reported that aberrant activation of the cGAS-STING pathway contributes to the development of fibrotic lung diseases [112]. Xu and colleagues discovered that Ficolin B, carried by exosomes from alveolar macrophages, exacerbates bleomycin-induced lung injury and fibrosis by promoting ferroptosis through the cGAS/STING signaling pathway [113]. The Nrf2/HO-1 signaling pathway is essential in assisting cells to counteract oxidative stress, inflammation, and the activation of detrimental signaling pathways, thus impeding the advancement of PF [114]. Moreover, this pathway is implicated in regulating ferroptosis. Research indicates that dihydroartemisinin (DHA) regulates the Nrf2/HO-1 pathway to mitigate cellular ferroptosis, subsequently attenuating radiation-induced lung injury and the extent of PF [115]. Similarly, *Tripterygium wilfordii* Hook.f. demonstrates potential in ameliorating paraquat-induced lung injury and fibrosis by mitigating oxidative stress and ferroptosis through the Nrf2/HO-1 pathway, which further underscores the crucial regulatory role of this pathway in the PF progression [116].

However, the pathogenesis of PF is intricate and multifaceted. Further research is warranted to explore how other specific pathways implicated in PF pathogenesis, such as the PI3K/Akt signaling pathway, WNT/β-catenin signaling pathway, JAK/STAT signaling pathway, and AMPK signaling pathway, interact with ferroptosis in regulating the development of PF.

## Endogenous Inhibitory System of Ferroptosis

**Fig. 5:**
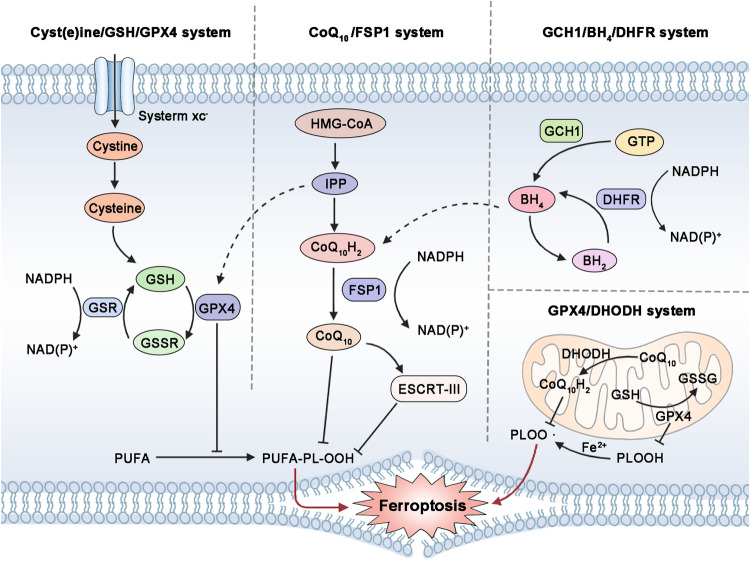
Endogenous ferroptosis inhibitory systems. Lipid peroxidation of membrane phospholipids can be eliminated by several endogenous ferroptosis inhibitory systems, such as Cyst(e)ine/GSH/GPX4 system, NADPH/FSP1/CoQ10 system, GCH1/BH4/DHFR system, and GPX4/DHODH system.

Cells have evolved several endogenous antioxidant systems to promptly counteract the instability of intracellular iron and ROS, mainly including the Cyst(e)ine/GSH/GPX4 system, NADPH/FSP1/CoQ10 system, GCH1/BH4/DHFR system, GPX4/DHODH system, and other defense systems [73, 88, 117–119] (Fig. 5).

### Cyst(e)ine/GSH/GPX4 system

The GSH/GPX4 system functions as the primary defense mechanism against cellular oxidative stress and is indispensable for suppressing ferroptosis [120]. GSH, a tripeptide consisting of glutamate, cysteine, and glycine, is biosynthesized by glutamate-cysteine ligase (GCL) and GSH synthase (GSS). System xc^-^ facilitates the intracellular uptake of cystine and catalyzes its conversion into cysteine, a crucial precursor for GSH synthesis and peroxidation reduction [20, 120]. However, erastin can block cysteine uptake and deplete GSH levels, leading to ferroptosis induction. The enzyme GPX4 is responsible for catalyzing the reduction of phospholipid hydroperoxides to non-toxic phospholipids, and GSH serves as a substrate for GPX4 [121]. Moreover, the activity of GPX4 is directly influenced by the absence of GSH [122]. It is worth noting that the primary strategy to induce ferroptosis currently involves either genetic knockout or pharmacological inhibition of GPX4 [123].

### NAD(P)H/FSP1/CoQ10 system

In recent years, several non-GPX4-dependent pathways that inhibit ferroptosis have been gradually elucidated. FSP1 has been identified as the second independent system inhibiting ferroptosis in addition to GPX4 [124]. Coenzyme Q10 (CoQ10), also referred to as ubiquinone, primarily facilitates electron transfer from complexes I and II to complex III in the mitochondrial electron transport chain. Its reduced form, ubiquinol (CoQ10H2), acts as a potent lipophilic antioxidant by scavenging free radicals and preventing lipid peroxide formation [125]. FSP1 can impede ferroptosis through NAD(P)H-mediated reduction of CoQ10 and Vitamin K-dependent elimination of lipid peroxidation [126]. The endosomal sorting complex required for transport-III (ESCRT-III) functions as a membrane repair system that mends plasma membrane rupture induced by PUFA-PL-OOH. In certain cases, ESCRT-III also restricts ferroptotic progression in a FSP1-dependent manner [127, 128]. Therefore, FSP1/CoQ10 can synergistically cooperate with the GSH/GPX4 pathway to inhibit ferroptosis [123, 126].

### GCH1/BH4/DHFR system

A recent genome-wide CRISPR activation screen has identified GTP-cyclic hydrolase 1 (GCH1), a potent antioxidant that scavenges free radicals (RTA), as the key enzyme responsible for tetrahydrobiopterin (BH4) synthesis. Notably, the GCH1/BH4 axis has been recognized as an alternative GPX4-independent pathway to inhibit ferroptosis caused by GPX4 deficiency [129, 130]. However, the recycling process of BH4 requires the participation of dihydrofolate reductase (DHFR). Deficiency of DHFR may increase cellular vulnerability to ferroptosis [131]. Moreover, BH4 acts as an antioxidant by converting phenylalanine into tyrosine and facilitating CoQ10 synthesis. Oral administration of sepiapterin, a precursor to BH4, has demonstrated elevated plasma levels of BH4 in rats and alleviated BLM-induced PF [132].

### GPX4/DHODH system

Mitochondria play a crucial role in iron metabolism and the generation of ROS [133–135]. Dihydroorotate dehydrogenase (DHODH) and mitochondrial GPX4 are responsible for converting CoQ10 to ubiquinol and detoxifying lipid peroxide within mitochondria, thereby contributing to the regulation of ferroptosis [136]. However, the inhibitory effect of DHODH on ferroptosis is relatively subtle. Only at high concentrations does DHODH inhibitor exhibit significant sensitization towards ferroptosis, while also effectively suppressing FSP1 activity [137]. Further investigations are warranted to elucidate the precise role of mitochondria and unravel the underlying molecular mechanisms implicated in ferroptosis [138, 139].

### Other defense mechanisms

Other defense mechanisms include the nuclear factor erythroid 2 related factor 2 (Nrf2)-mediated pathway, the transsulfuration pathway, and the mTOR signaling pathway [81]. Nrf2 binds to and activates antioxidant response elements (ARE), promoting antioxidant gene expression and transcription of FSP1 [140]. Anandhan et al. report that Nrf2 regulates intracellular LIP levels and reduces ferroptosis by controlling ferritin synthesis and degradation [141]. As a downstream target of Nrf-2, oxygenase-1 (HO-1) represents one of the most crucial cellular adaptation mechanisms to oxidative stress. Under physiological conditions, its activation aids in scavenging ROS and protecting cells from oxidative stress. However, hyperactivation of HO-1 leads to increased intracellular ROS levels and iron accumulation [142]. Increasing evidence implicates that overexpression of HO-1 triggers ferroptosis through enhanced iron accumulation and lipid peroxidation [143]. Nevertheless, revealing the underlying mechanism of Nrf2 agonists or HO-1 inhibitors in the treatment of PF warrants further investigation. Xue and colleagues found that the transsulfuration pathway provides a novel mechanism for cells to obtain essential components for GSH synthesis by degrading methionine into cysteine to maintain GPX4 activity (81). Ferroptosis can be induced by depriving cells of methionine or inhibiting the transsulfuration pathway [144, 145]. Furthermore, cysteine not only serves as a precursor for GSH synthesis but also contributes to coenzyme A (CoA) production which may act as a potential substrate for CoQ10 synthesis [146]. Recent mechanistic work revealed that ferroptosis could inhibite by mTOR, which in turn upregulates the expression of Sterol Regulatory Element Binding Protein 1 (SREBP1), leading to monounsaturated fatty acid (MUFA) production [147]. According to Cheng’s study, synovial macrophages release Semaphorin 5 A activating PI3K/AKT/mTOR signaling, thereby effectively preventing the occurrence of ferroptosis [148].

## Strategies For Targeted Ferroptosis in PF

Aberrant iron homeostasis has been demonstrated to be a critical mechanism underlying the progression of PF. The accumulating evidence indicates that targeting ferroptosis represents a promising novel therapeutic strategy for managing PF [110, 149]. However, the precise mechanisms underlying the initiation and execution of ferroptosis remain elusive, as well as the downstream signaling molecules and pathways involved in mediating the progression of PF [150]. Currently, several strategies have been proposed to impede PF progression by modulating the ferroptosis-related pathway: iron chelation, prevention of lipid peroxidation, elimination of lipid peroxides, and activating endogenous ferroptosis inhibition system (Table 1).

**Table 1:** Potential therapeutic drugs targeting ferroptosis for PF.

Therapeutic agents	Targeting molecule	Potential Mechanism	Application	Ref.
DFO	Iron chelator	Inhibit fenton reaction and mitochondrial ROS production	Attenuate pulmonary epithelial cell death and fibrosis induced by cigarette smoke	[25]
DFP	Iron chelator	Inhibit fenton reaction and iron-containing lipid oxygenases	Attenuate PF in transfusion-dependent patients with thalassemia major	[153]
CQ	Iron chelator	Inhibit fenton reaction and alleviate inflammatory responses	Attenuates PF induced by BLM and PQ	[108]
Ciclopirox	Iron chelator	Inhibit fenton reaction	Attenuate cystic fibrosis lung infections	[155]
Rosiglitazone	ACSL4 inhibitor	Upregulate PTEN and decrease TGF-β	Attenuate PF induced by PQ and BLM	[157, 158]
pioglitazone	ACSL4 inhibitor	Activate PPAR-γ to remedy fatty acid oxidation	Attenuate cardiac fibrosis	[159]
Troglitazone	ACSL4 inhibitor	Inhibit synthesis of TGF-β and fibroblast proliferation and differentiation	Attenuate peritoneal fibrosis and PF	[161, 162]
Empagliflozin	SGLT2 inhibitor	Inhibit ferroptosis via Sesn2/AMPK/Nrf2/HO-1 pathway	Attenuate PF	[164]
Sepiapterin	RTA	Increase BH4 levels in plasma	Attenuate PF induced by BLM	[132]
Ferrtatin-1	RTA	Inhibit lipid peroxidation	Attenuate silicosis fibrosis	[167]
Liproxstatin-1	RTA	Inhibit lipid peroxidation	Attenuate RILF	[182]
Se-Met	Selenium supplement	Inhibit cGAS/STING/NF-κB pathway.	Attenuate lung epithelial cells senescence	[174]
Ebselen	Selenium supplement	Decrease oxidized DAG	Attenuates PF induced by BLM	[175]
Allosteric GPX4 activators	GPX4 agonist	Enhance the acticity of GPX4	Unkown	[177]
NAC	GSH precursor	Increase pulmonary GSH levels	Attenuate PF induced by BLM	[178, 179]
β-mercaptoethanol	Reductant	Promote system xc^-^ uptake of cysteine	Suppresse human lung fibroblast proliferation	[183]
Sulforaphane	Nrf-2 agonist	Decrease 4-HNE levels	Attenuate PF induced by BLM	[127]
DHQ	flavonoid	Inhibit ferritin autophagy	Attenuate Silicosis	[27]
Fraxetin	NCOA4	Form stable binding with NCOA4 and reduces ferritin autophagy	Attenuate PF induced by BLM	[150]
Baicalein	Arachidonic Acid inhibitor	Inhibit GPX4 degradation	Attenuate PF	[185–188]
Zileuton	LOX inhibitor	Inhibit LOXs induced lipid peroxidation	Attenuate IPF	[189]
Ficolin B	Unkown	Promote ferroptosis through the cGAS/STING signaling pathway	Attenuate PF induced by BLM	[113]
Dihydroartemisinin	Unkown	Regulate the Nrf2/HO-1 pathway to mitigate cellular ferroptosis	Attenuate radiation-induced lung injury and PF	[115]
*Tripterygium wilfordii* Hook.f.	Unkown	Mitigate oxidative stress and ferroptosis through the Nrf2/HO-1 pathway	Attenuate paraquat-induced lung injury and fibrosis	[116]
ELA-32	mTOR agonist	Activate APJ-Akt-mtor-P70S6K signaling	Attenuate myocardial fibrosis	[190]
Liraglutide	Unkown	Elevate the expression of SLC7A11 and the Nrf2/HO-1/GPX4 signaling pathway	Attenuate liver fibrosis	[192]
Roxadustat (FG-4592)	HIF inhibitor	Inhibit ferroptosis via Akt/GSK-3β/Nrf2 Pathway	Attenuate kidney fibrosis induced by folic acid	[193]
Zinc protoporphyrin	HO-1 inhibitor	Inhibit HO-1 mediated mitochondrial dysfunction	Attenuate PF induced by PM2.5	[28]

*RTA* radicals trap antioxidants, *PF* Pulmonary fibrosis, *RILF* Radiation-induced lung fibrosis, *BLM* Bleomycin, *DFO* Defetoxamine, *DFP* Deferiprone, *CQ* Chloroquine, *PQ* Paraquat, *ACSL4* Long chain acyl-CoA synthetase 4, *PTEN* Phosphatase and tensin homolog, *TGF-β* Transforming Growth Factor-β, *PPAR-γ* proliferator-activated receptor γ, *DAG* diacylglycerol, *Se-Met* Selenomethionine, *LOX* lipoxygenase, *IPF* Idiopathic pulmonary fibrosis. *GPX4* Glutathione peroxidase 4, *Nrf-2* Nuclear factor erythroid 2-related factor 2, 4-*HNE* 4-Hydroxy-2-nonenal, *NAC* N-acetylcysteine, *GSH* Glutathione, *ELA-32* Elabela −32, *mTOR* mechanistic target of rapamycin, *HIF* Hypoxia-inducible factor, *GSK-3β* Glycogen synthase kinase-3, *HO-1* Heme oxygenase-1, *PM2.5* particulate matter 2.5, *NCOA4* Nuclear receptor coactivator 4, *RIPK* receptor interacting protein kinase, *SIRT1* Sirtuin-1, *SLC7A11* Solute carrier family 7 membrane 11.

### Iron chelation

Iron participates in the Fenton reaction, leading to the generation of ROS [89, 151]. Additionally, it serves as an essential component of various enzymes, such as lipoxygenases (LOX) and NOX, which play a pivotal role in catalyzing enzymatic reactions [152]. The precise mechanism by which iron contributes to lipid peroxidation and ferroptosis remains uncertain; however, iron chelation has proven effective in blocking lipid peroxidation and associated ferroptosis. Clinically used iron chelators such as Deferoxamine (DFO), deferiprone (DFP), and deferasirox (DFS) are employed for treating transfusion iron overload in non-transfusion-dependent thalassemia patients [153, 154]. Evidence shows that DFO and chloroquine (CQ) attenuate the Fenton reaction by sequestering excessive iron ions, thereby mitigating both BLM-induced lung injury and cigarette smoke-induced PF in mice models [25, 108]. Ciclopirox also exhibits affinity for binding to iron ions and is widely utilized clinically for antifungal infections. Kadiam et al. demonstrated that ciclopirox effectively inhibits the progression of cardiac fibrosis through blocking fibroblast activation [155]. Although ciclopirox displayed promising results against cystic fibrosis lung infections during in vitro experiments, its potential cytotoxicity should be considered [12]. However, clinical studies investigating the efficacy of iron chelators for treating PF are currently lacking.

### Prevention of lipid peroxidation

ACSL4 inhibitors, such as rosiglitazone, troglitazone, and pioglitazone, have been utilized to inhibit lipid peroxidation and esterification of PUFAs in a lipoxygenase-dependent manner [80]. Additionally, they act as agonists for PPAR to upregulate the Nrf2 antioxidant response [156]. Rosiglitazone exhibits remarkable efficacy in attenuating the progression of PF induced by BLM and PQ [157, 158]. In particular, pioglitazone activates PPAR-γ and attenuates fatty acid oxidation to halt myocardial fibrosis and heart failure [159]. Huang et al. combined pioglitazone with iron oxide nanoparticles for efficient delivery of MSCs to fibrotic AECs and restoration of damaged mitochondria in fibrotic tissue [160]. Among the thiazolidinedione family members, troglitazone is considered the most potent inhibitor of ferroptosis due to its intrinsic antioxidant activity driven by the tryptophanol portion of its structure [80]. Studies have demonstrated that troglitazone inhibits TGF-β synthesis [161] as well as proliferation and differentiation of lung fibroblasts induced by TGF-β [162]. An alternative approach to mitigating lipid peroxidation involves the utilization of exogenous MUFAs [163]. While the precise mechanism remains incompletely elucidated, exogenous MUFAs have demonstrated efficacy in impeding ferroptosis and restricting lipid peroxidation on the plasma membrane. This inhibitory effect may be attributed to competitive interactions between MUFAs and PUFAs [18]. Interestingly, a diet rich in MUFA was found to inhibit pirfenidone-induced adverse reactions and enhance its efficacy in the treatment of IPF [164]. Empagliflozin, a sodium-glucose cotransporter 2 (SGLT2) inhibitor, exhibits potential protective effects in PF by preventing lipid peroxidation. Current evidence indicates that empagliflozin demonstrates a promising ability to protect against bleomycin-induced PF in rats by inhibiting ferroptosis via modulating the Sesn2/AMPK/Nrf2/HO-1 signaling pathway.

### Clearance of lipid peroxides

Several free radical trapping antioxidants (RTAs), such as α-tocopherol (the active form of vitamin E), ferrostatin-1 (Fer-1), and liproxstatin-1 (Lip-1), have demonstrated efficacy in scavenging lipid peroxides for the treatment of PF [24, 156]. These RTAs exhibit potent scavenging activity towards phenols and aromatic amines, which are characterized by relatively weak OH and NH bonds, respectively [165]. Of note, Fer-1 is a widely utilized small molecule inhibitor of ferroptosis in laboratory settings. It has been reported to upregulate GPX4 expression and impede the progression of PF [166–168]. However, its unfavorable pharmacokinetic profile renders it unsuitable for clinical application [20].

### Activating endogenous ferroptosis inhibition system

Aside from the aforementioned strategies, activating the endogenous ferroptosis inhibitor system may also inhibit PF progression. Selenium, a trace element, plays a crucial role in regulating cellular redox homeostasis during oxidative stress [169, 170]. It serves as an integral component of selenocysteine and is situated within the catalytic site of antioxidant enzymes such as GPXs, thioredoxin reductases (TXNRDs), and selenoprotein P (SELENOP) [171, 172]. Given that GPX4 is a selenoprotein, supplementation with selenium can potentiate its activity [173]. Wang et al. revealed that supplementation with selenomethionine (Se-Met) mitigates PM2.5-induced lung epithelial cell senescence [174]. Conversely, PM2.5 significantly exacerbates the initial PF process by triggering ferroptosis [28]. Ebselen, an organic selenium-based ferroptosis inhibitor, has the ability to attenuate BLM-induced PF in mice [175]. BS1801, an analog of ebselen, is currently undergoing clinical trials for the treatment of liver fibrosis and PF [176]. Additionally, certain novel GPX4 variant activators may be employed as inhibitors of ferroptosis [177]. N-acetylcysteine (NAC), a precursor of glutathione (GSH), has been found to suppress 4-HNE-induced ROS production and restore GSH levels [178, 179]. High-dose NAC inhalation therapy shows promise in rectifying the oxidant-antioxidant imbalance observed in lung tissue of IPF patients, thereby ameliorating fibrotic progression [180]. β-mercaptoethanol functions as a reducing agent that facilitates cysteine uptake for maintaining optimal GSH levels [181, 182]. Studies have demonstrated that β-mercaptoethanol can inhibit the proliferation of human lung fibroblasts by modulating ferroptosis-related pathway [183]. Nrf2 agonists also exhibits anti-fibrotic effects. Sulforaphane mitigated BLM-induced PF by reducing the level of 4-HNE through activation of Nrf2. Li and colleagues demonstrated that liproxstatin-1 attenuates radiation-induced PF via activation of the Nrf2 pathway [184]. It has been reported that Dihydroquercetin (DHQ) inhibited ferritin autophagy and decreased ferrous iron in unstable intracellular iron pools by downregulating microtubule-associated protein 1 A/1B-light chain 3 (LC3) and upregulating ferritin heavy chain 1 (FTH1), as well as nuclear receptor co-activator 4 (NCOA4), in activated HBE cells for silicosis treatment [27]. Evidence indicates that fraxetin, extracted from Fraxinus rhynchophylla, inhibits ferritin autophagy by forming a stable complex with NCOA4 [150]. Baicalein and esculetin are inhibitors of arachidonic acid metabolism that alleviate PF by inhibiting lipid peroxidation [185–187]. Xie et al. reported that baicalein exhibited potent inhibition against ferroptosis through binding to Fer-1, Lip-1, DFO, and β-mercaptoethanol. Additionally, it hindered GPX4 degradation mediated by erastin [188]. Recently, it has been discovered that 5-lipoxygenase (5-LOX) is involved in the process of lipid peroxidation. Zileuton, a 5-LOX inhibitor approved by the FDA and formerly employed in the treatment of asthma, is presently under evaluation for clinical trials in the management of PF [189].

Aside from the molecules mentioned above, several other compounds with anti-ferroptotic properties have demonstrated antifibrotic effects in various organs besides the lungs, and hold promise for potential therapeutic application in PF. Elabela-32 (ELA-32), an mTOR agonist, has demonstrated efficacy in mitigating myocardial infarction and attenuating myocardial fibrosis in rat models [190]. Furthermore, it has exhibited the ability to reverse TGF-β1-induced epithelial-mesenchymal transition (EMT) in human peritoneal mesothelial cells (HPMC) (123) and pulmonary vascular remodeling induced by pulmonary arterial hypertension (PAH) [191]. Liraglutide is a glucagon-like peptide-1 (GLP-1) receptor that elevates the expression of SLC7A11 and activates the Nrf2/HO-1/GPX4 signaling pathway to attenuate liver fibrosis in db/db mice [192]. Roxadustat (FG-4592), an inhibitor of prolyl hydroxylase of hypoxia-inducible factor (HIF), mitigates folic acid-induced kidney fibrosis via the Akt/GSK-3β/Nrf2 pathway [193]. Intriguingly, necrostatin-1 (Nec-1), a widely used inhibitor of necroptosis, has been suggested to concurrently inhibit ferroptosis [194]. Further investigation is imperative to comprehensively elucidate the underlying mechanism governing this interplay between necroptosis and ferroptosis in PF. Notably, it should be mentioned that Nec-1 exhibits a more favorable pharmacokinetic profile compared to Fer-1. Therefore, Nec-1 may possess a better translational potential than Fer-1 due to its relatively well-tolerated maximum tissue concentration [195].

In conclusion, further investigation is warranted to elucidate the underlying pathways and regulatory mechanisms associated with ferroptosis in PF. Preliminary findings from numerous preclinical studies suggest that ferroptosis inhibitors exhibit efficacy in various PF animal models [196]. Subsequent research should focus on developing more potent ferroptosis inhibitors, aiming to advance these compounds into clinical trials.

## Prognostic and diagnostic implications of ferroptosis-related biomarkers in PF

The challenge of reversing fibrosis remains a significant obstacle in this field, highlighting the critical importance of early diagnosis of PF [197–199]. However, reliable and precise diagnostic and prognostic biomarkers for PF are still lacking. Therefore, it is imperative to gain an in-depth understanding of the molecular mechanisms underlying PF development and identify novel biomarkers. Recent findings have shed new light on the diagnostic and prognostic significance of ferroptosis-related biomarkers in PF (Table 2).

**Table 2:** Potential ferroptosis-related biomarkers for PF.

Category	Biomaker	Location	Reference
Lipid peroxidation	8-Isoprostane	BALF and serum	[200]
	4-HNE	Fibroblast and BALF	[201]
	MDA	BALF, plasma, and serum	[60]
	Ethane	Breath samples	[61]
	GSH	Sputum and BALF	[60, 202]
	GSTP	BALF	[203]
	SOD	BALF, serum, and fibrotic tissues of PF lung	[203, 204]
Ferroptosis	N-ras	BALF	[206, 207]
	ACO1	BALF	[208]
	ENPP2	BALF	[206]
	MUC1	BALF	[210, 211]
	ZFP36	BALF and Lung tissue biopsies of PF	[206]
	BH4	Plasma	[132]
	SLC7A11	Primary human lung fibroblasts	[209]
	TfR1	BALF	[212, 213]
	DMT1	BALF	[212]
	ferritin-1	BALF	[214]
	Nrf2	Lung tissue, BALF, and blood samples	[215]
	GPX4	BALF	[59]

*PF* Pulmonary fibrosis, *4-HNE* 4-hydroxy-2-nonenal, *BALF* bronchoalveolar Lavage Fluid, *NOX-4* NADPH oxidase 4, *GSH* glutathione, *GSTP* glutathione S-transferase P, *MDA* malondialdehyde, *SOD* superoxide dismutase, *ACO-1* aconitase 1, *MUC1* Mucin 1, *ENPP2* ctonucleotide pyrophosphatase/phosphodiesterase 2, *ZFP36* zinc finger protein 36, *BH4* tetrahydrobiopterin, S*LC7A11* Solute Carrier Family 7 Member 11, *TfR1* transferrin receptor 1, *DMT1* divalent metal transporter 1, *Nrf2* nuclear factor erythroid 2-related factor 2, *GPX4* glutathione peroxidase 4.

Lipid peroxidation is a central molecular event in the pathogenesis of PF, and its key molecules have the potential to serve as biomarkers for early disease diagnosis. Elevated levels of specific lipid peroxidation products, such as 8-Isoprostane [200], 4-HNE [201] and MDA [60], have been identified in BALF from IPF patients. Ethane, a volatile organic compound, is a by-product of lipid peroxidation. The concentration of ethane in exhaled breath reflects the level of oxidative stress in the body, making it a potential non-invasive biomarker for early PF diagnosis [61]. Additionally, antioxidants hold diagnostic and prognostic value for PF patients. Reduced levels of GSH [60, 202], GSTP [203] and SOD [203, 204] have been observed in both BALF and serum samples from PF patients, indicating an imbalanced status of lipid peroxidation in PF progression.

Alterations in ferroptosis-related genes (FRGs) have also been reported in PF patients and utilized for predicting disease progression. He and colleagues identified eight FRGs, including N-Ras, epithelial membrane protein 1 (EMP1), MYC, Mucin 1 (MUC1), and GABA Type A Receptor Associated Protein Like 1 (GABARAPL1) in BALF of IPF patients [205]. Furthermore, they have successfully developed predictive models to establish these genes as prognostic biomarkers for IPF [205]. Another study conducted a comparison of five FRGs namely aconitase 1 (ACO1), N-Ras, MUC1, zinc finger protein 36 (ZFP36), and ectonucleotide pyrophosphatase/phosphodiesterase 2 (ENPP2), retrieved from the FerrDb database that have potential applications in the diagnosis, treatment, or prognosis of IPF [206]. Among them, N-Ras is implicated in fibrosis through its involvement in TGF-β1-induced proliferation, collagen and fibronectin synthesis [207], and is associated with an unfavorable prognosis in IPF patients [206]. The expression of ACO1, which regulates cellular iron levels, is also downregulated in IPF patients [208]. SLC7A11 is an integral component of the system xc^-^ and one of the extensively studied biomarkers associated with ferroptosis. Reduced levels of SLC7A11 have been observed in fibroblasts associated with IPF, which exhibit a senescent phenotype [209]. The stability of system xc^-^ is enhanced by the formation of a complex between MUC1 and CD44 [210]. Deficiency of MUC1 exacerbates fibrosis progression in silicosis murine models, and MUC1 levels could be used to predict the severity of PF [211]. In addition, several genes involved in iron metabolism, including TfR1 [212, 213], DMT1 [212] and ferritin-1 [214], exhibit aberrant expression in BALF and could be helpful in assessing the prognosis of PF patients. As previously mentioned, activators of Nrf2 demonstrate robust antifibrotic effects and effectively reduce PF both in vivo and in vitro. Evidence suggests that Nrf2 expression was decreased in lung tissue, BALF, and blood samples obtained from mice with PF [215]. Additionally, Nrf2 knockout lead to the formation of fibrotic tissues, indicating that Nrf2 is a potential ferroptosis-related therapeutic target and biomarker for evaluating the severity of PF [215]. Among GPX isoforms, GPX4 is a distinctive antioxidant enzyme capable of directly reducing phospholipid hydroperoxides. Importantly, levels of GPX4 were found to decrease in an in vivo model of bleomycin-induced PF [59]. The reduction of GPX4 leading to lipid peroxidation may play a role in myofibroblast differentiation and the development of PF [59]. Therefore, GPX4 represents a promising detection marker and therapeutic target for addressing PF.

## Conclusion and perspective

PF is a fatal chronic lung disease, and recent studies have shed light on the involvement of lipid peroxidation and ferroptosis in its pathogenesis. The newly developed ferroptosis-associated therapeutic strategy possesses considerable promise for PF treatment. In this review, we systematically summarize current discoveries regarding the role of ferroptosis in the pathogenesis of PF and discuss potential biomarkers and drugs for anti-fibrotic therapeutic strategies. Indeed, a comprehensive understanding of the significance of lipid peroxidation and ferroptosis in PF opens up avenues for therapeutic interventions. Recently, promising therapeutic strategies have emerged, such as employing iron chelators, antioxidants, and preventing lipid peroxidation to inhibit ferroptosis.

Nevertheless, the translation of these findings into clinically effective therapies poses challenges. Firstly, one of the primary obstacles is the absence of reliable biomarkers in PF patients that can accurately indicate the extent of lipid peroxidation and ferroptosis, making it difficult to assess disease severity and progression. In fact, the regulation of peroxidation and ferroptosis involves a complex network of biochemical pathways that remain poorly understood in many aspects. Despite the investigation of certain biomarkers associated with ferroptosis, their practical application remains limited. Accordingly, the application of innovative methods including single-cell omics, metabolomics, high-resolution imaging, and liquid biopsy could facilitate the identification of novel ferroptosis-related biomarkers in the biological process of PF. Secondly, the clinical applicability of ferroptosis inhibitors is impeded by their unfavorable pharmacokinetic profile. For instance, Fer-1, similar to other hydrophobic drug candidates, is unsuitable for clinical development because of its limited capacity to traverse the blood-brain barrier (BBB) and its poor water solubility [216]. Moreover, the long-term effects, safety profiles, drug interactions, and patient-specific application of ferroptosis inhibitors remain unknown, warrants further studies. Therefore, during the process of modifying the structure of ferroptosis inhibitors, careful consideration should be given to their pharmacokinetic properties in order to enhance their bioavailability. Thirdly, inhibiting ferroptosis as a therapeutic approach may have potential side effects since this RCD mechanism also plays an essential role in normal physiological processes. Improper management of ferroptosis induction could lead to deleterious effects on adjacent normal tissue to fibrotic tissue. Fortunately, nanoparticles carrying chemicals or biological materials may assist in overcoming the limitations, which combine ferroptosis inducers and certain antibodies on the surface of nanoparticles and enhance the targeting capacity towards fibrotic tissues while minimizing potential side effects.

Overall, it is evident that ferroptosis has yet to disclose all its secrets involved in the pathologic process of PF. Despite the existing challenges, a deeper comprehending of the regulatory mechanism of ferroptosis and its contribution to PF holds immense potential for identifying reliable biomarkers and efficacious therapeutic interventions. In particular, the combination of ferroptosis inhibitors with additional anti-fibrotic drugs such as pirfenidone and nidazanib will offer novel prospects for the treatment of PF. We believe that ferroptosis-focused studies will open up new perspectives for the diagnosis and treatment of PF.

## Data Availability

All data generated or analyzed during this study are included in this published article.
